# Chronic Kidney Disease-Associated Inflammation Increases the Risks of Acute Kidney Injury and Mortality after Cardiac Surgery

**DOI:** 10.3390/ijms21249689

**Published:** 2020-12-18

**Authors:** Angela Casas, Adrián Mallén, Arnau Blasco-Lucas, Fabrizio Sbraga, Jordi Guiteras, Núria Bolaños, Esther Castaño, Joan Torras, Josep M. Cruzado, Estanislao Navarro, Miguel Hueso

**Affiliations:** 1 Experimental Nephrology Lab, Institutd’Investigació Biomèdica de Bellvitge-IDIBELL, L’Hospitalet de Llobregat, 08907 Barcelona, Spain; angelita_2121@hotmail.com (A.C.); amallen@idibell.cat (A.M.); jguiteras@idibell.cat (J.G.); nbolanos@idibell.cat (N.B.); 2 Department of Cardiac Surgery, Hospital Universitari de Bellvitge, L’Hospitalet de Llobregat, 08907 Barcelona, Spain; arnaulasco@gmail.com (A.B.-L.); sbraga@bellvitgehospital.cat (F.S.); 3 Biology Unit, CCiT-Universitat Barcelona, L’Hospitalet de Llobregat, 08907 Barcelona, Spain; mcastano@ub.edu; 4 Department of Nephrology, Hospital Universitari de Bellvitge, L’Hospitalet de Llobregat, 08907 Barcelona, Spain; 15268jta@comb.cat (J.T.); jmcruzado@bellvitgehospital.cat (J.M.C.)

**Keywords:** chronic kidney disease, cardiac surgery–associated acute kidney injury, coronary artery diseases, inflammation, CD14^++^CD16^+^ monocytes, hsa-miR-30a-5p

## Abstract

Cardiovascular mortality increases with decreasing renal function although the cause is yet unknown. Here, we have investigated whether low chronic inflammation in chronic kidney diseases (CKD) could contribute to increased risk for coronary artery diseases (CAD). Thus, a prospective case–control study was conducted in patients with CAD and CKD undergoing coronary artery bypass graft surgery with the aim of detecting differences in cardiovascular outcomes, epicardial adipose tissue volume, and inflammatory marker activity associated with renal dysfunction. Expression of membrane CD14 and CD16, inflammatory cytokines and chemokines, mitogen-activated protein (MAP) kinases and hsa-miR-30a-5p were analyzed in peripheral blood mononuclear cells (PBMCs). Epicardial fat volume and tissue inflammation in perivascular adipose tissue and in the aorta were also studied. In the present study, 151 patients were included, 110 with CAD (51 with CKD) and 41 nonCAD controls (15 with CKD). CKD increased the risk of cardiac surgery–associated acute kidney injury (CSA-AKI) as well as the 30-day mortality after cardiac surgery. Higher counts of CD14^++^CD16^+^ monocytes were associated with vascular inflammation, with an increased expression of *IL1β*, and with CKD in CAD patients. Expression of hsa-miR-30a-5p was correlated with hypertension. We conclude that CKD patients show an increased risk of CSA-AKI and mortality after cardiovascular surgery, associated with the expansion of the CD14^++^CD16^+^ subset of proinflammatory monocytes and with *IL1β* expression. We propose that inflammation associated with CKD may contribute to atherosclerosis (ATH) pathogenesis.

## 1. Introduction

Atherosclerotic vascular disease is more common and severe in patients with chronic kidney disease (CKD) and increases their mortality risk [1,2]. However, this increased risk shows significant variations among different studies for reasons that are yet unclear but might be related to a background of systemic and/or local vascular inflammation known to be critical in atherosclerosis progression [3,4,5,6]. Consequently, the identification of factors linking CKD and coronary artery diseases (CAD) should improve the current understanding of how an impaired kidney function leads to higher cardiovascular risks as well as to identify novel therapeutic targets.

A link between cellular immunity and CAD has been proposed with a key role of monocytes and macrophages [7,8,9]. However, monocytes are a heterogeneous population with a differential functional role [10] and human monocytes have been classified based on their profiles of CD14 (LPS co-receptor) and CD16 (low-affinity FCγ receptor type III) expression into three subtypes: “classical” CD14^++^/CD16^−^, “intermediates” CD14^+^CD16^++^, and “non-classical” CD14^++^/CD16^+^ monocytes [11]. In this sense, it is important to notice that the definition of monocyte subpopulations is challenging since expression of the CD16 marker is continuous, and there is as yet no consensus in the demarcation of the different monocyte subsets that could facilitate the development of functional studies [12,13]. In addition, there are very few clinical studies investigating the differential role of monocyte subsets in CAD. In one of these, higher cell counts of CD14^++^CD16^+^ monocytes predicted cardiovascular events [9]. Interestingly, addition of C-reactive protein (CRP) values higher than 2 mg/L to CD14^++^CD16^+^ counts highlighted a subgroup of patients with the worst outcome [9]. Furthermore, in CKD patients, CD14^++^CD16^+^ monocytes were shown to contribute to an accelerated atherosclerosis (ATH) with higher rates of cardiovascular events [7,14]. All these data suggest that the different monocyte subpopulations might have different biological functions in ATH progression.

We hypothesized that CAD patients with CKD present different pathogenic profiles than patients with normal renal functions and that profiling monocyte subpopulations could be useful to identify their distinct proinflammatory environments.

## 2. Results

### 2.1. CKD Is Associated with Increased Risk of Cardiac Surgery–Associated Acute Kidney Injury (CSA-AKI) and 30-Day Mortality after Cardiac Surgery Cardiovascular Outcome

A total of 151 patients were included in the study, 110 patients had CAD (85 male) from which 51 also suffered CKD (stages 3 to 5), and another set of 41 patients were nonCAD controls (30 male) from which 15 patient suffered CKD. In all, 38 patients underwent combined coronary artery bypass graft (CABG) with valve replacement surgery. Demographics are shown in Table 1. Patients with CAD and CKD had a higher systolic blood pressure (129 ± 21 mmHg) than patients of CAD with normal renal function (121 ± 20 mmHg) or nonCAD controls (119 ± 11 mmHg; *p* = 0.02).

Ten out of the 110 CAD patients (9.1%) and three out of the 41 patients with nonCAD and valve surgery (7.3%) died after heart surgery. When considering the impact of renal function in the mortality of CABG surgery, we observed that, in the group with CAD, eight out of 51 patients with CKD (15.7%) died vs. two out of 59 patients with eGFR > 60 (3.4%) (*p* = 0.02). Furthermore, three out of 15 patients with heart valve replacement surgery and CKD died (20%) vs. none in the group of 26 patients without CKD (*p* = 0.017). These data support the impact of renal disease in the outcome of CABG surgery patients. In addition, the incidence of acute kidney injury (AKI) was 13 out of 51 (26%) in CAD + CKD, three out of 59 (5.1%) in CAD + nonCKD, four out of 15 (26.7%) in nonCAD + CKD, and two out of 26 (7.7%) in nonCAD + nonCKD patients (*p* = 0.008). Among the deceased patients, the incidence of AKI was 45.5% vs. 2.3% in surviving patients (*p* < 0.0001) suggesting that AKI increased the risk of mortality.

### 2.2. CKD Was Not Associated with an Increase in Total Volume of Epicardial Adipose Tissue (EATV)

**Figure 1:**
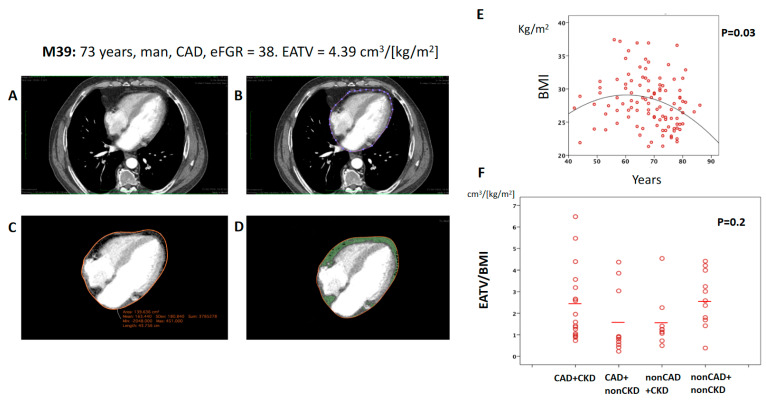
Measurement of the epicardial adipose tissue volume (EATV) in a non-contrast cardiac computed tomography. Measurements were performed as described in the Methods section number 4.4. Briefly, (**A**): representative image of a non-contrast cardiac computed tomography from the patient M39 of the current study, (**B**,**C**): tracking the contour of the pericardium (blue dots in B and continuous orange line in (**C**) to measure total epicardial adipose tissue volume in (**D**) using a semi-quantitative measurement of the epicardial fat volume (EFV) with Horos^TM^ (Horosproject.org). A minimum of 40 images were used and perivascular fat attenuation was within the threshold range of −30 to −250 Hounsfield units. The EFV, marked in green in the figure, was measured in cm^3^. (**E**): Dot-Blot showing an inverted U-shaped relation among BMI and patient age. (**F**): Dot-Blot showing EATV values (indexed to BMI) of the different groups of this study. Abbreviations: CAD = coronary artery disease, BMI = body mass index, EATV = epicardial adipose tissue volume, eGFR = estimated glomerular filtration rate, NonCKD = patients with eGFR > 60 mL/min.

EATV is an independent risk predictor for cardiovascular disease [15], so we analyzed its impact in a subgroup of 48 patients with available cardiac computed tomography. EATV was not different among patients in the CAD or nonCAD groups even when EATV was indexed to body mass index (BMI) (Figure 1A–D, shows how EATV was measured). However, a great variability was observed (Figure 1F), especially in the patients with valvular replacement, which showed higher scores in cases with severe aortic stenosis and dilation of ascendant aorta.

Aging, abdominal obesity, and heart hypertrophy have been reported as main predictors of high epicardial adiposity [16]. In our CAD cohort, BMI displayed an inverted U-shaped relation with age (*p* = 0.03, Figure 1E), and EATV indexed by BMI was correlated with gender since men presented higher EATV than women (2.50 ± 1.71 cm^3^/(kg/m^2^) in men, *n* = 21 vs. 0.91 ± 0.50 cm^3^/(kg/m^2^) in women, *n* = 7; *p* = 0.024) suggesting that age and gender are potential confounding factors. Additionally, in our patients EATV/BMI was not correlated with hypertension, creatinine, diabetes, hyperlipidemia, or with the size of adipocytes.

### 2.3. CKD Was Associated with the Increased Expression of Inflammatory Markers and with a Higher Proportion of Intermediate CD14^++^CD16^+^ Monocytes

**Figure 2:**
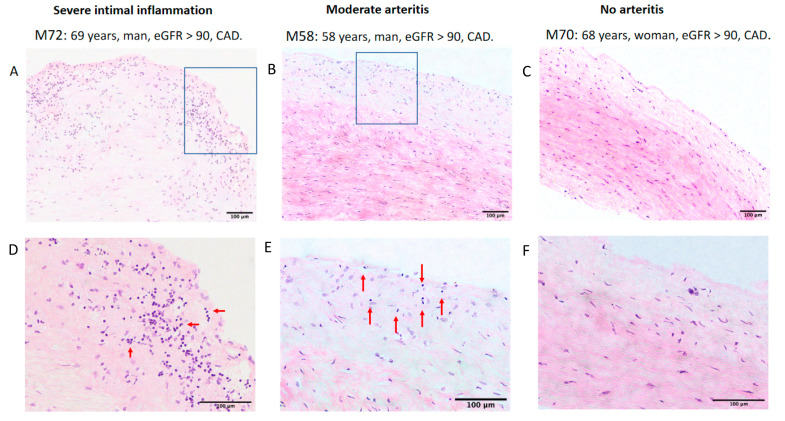
Severity of vascular inflammatory lesions as evaluated by a semi-quantitative scoring system based on the mononuclear infiltration of the intima. Shown are representative sample from three patients at two magnifications. Infiltrating mononuclear cells are indicated by red arrows. Frames in panels (**A–C**) represent the areas magnified in panels (**D–F**) for patients M72 and M58. For patient M70, both panels are from different areas because of the low number of infiltrating cells. The degree of inflammation was graded as “No arteritis” when <5 cell/field were observed, “mild arteritis” when there were 5–10 cells/field, “moderate arteritis” when there were 10–20 cells/field, and “severe intimal arteritis” when there were >20 cells/field. Bars show magnification. Abbreviations: CAD = coronary artery disease, eGFR = estimated glomerular filtration rate. Scale bar = 100 µm.

**Figure 3:**
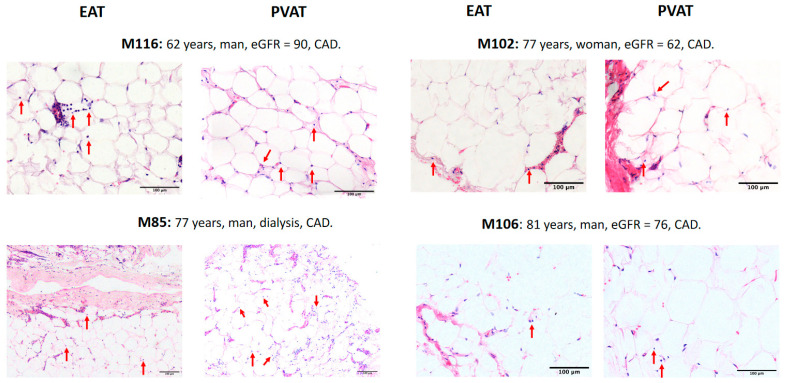
Severity of adipose tissue inflammation evaluated by a semi-quantitative scoring system based on the number of mononuclear infiltrated cells per adipocyte. Shown are representative samples of EAT and PVAT from four different patients. Infiltrating mononuclear cells are indicated by red arrows. The extent of adipose tissue inflammation was evaluated by scoring the number of infiltrating mononuclear cells per adipocyte cell. Scores were “none” (score = 0) when there were less than 2 inflammatory cells/adipocyte, “one” (greater than minimal), or “two” (when severe capillary hyperemia was observed). Bars show magnification. Abbreviations: CAD = coronary artery disease, EAT = epicardial adipose tissue, eGFR = estimated glomerular filtration rate, PVAT = perivascular adipose tissue. Scale bar = 200 µm.

A punch of the normal aorta and biopsies from the perivascular adipose tissue were obtained from 62 patients undergoing CABG surgery. Furthermore, aortic samples from seven patients from aortic valve heart surgery, and epicardial samples from other 16 patients from valve heart surgery were also taken. We next evaluated inflammatory markers in 41 aortic samples (Figure 2 shows a representative sample with the three different degrees of severity in the mononuclear infiltration of the intima used to score the lesions), as well as in 82 samples from epicardial adipose tissue (EAT) and other 58 samples from perivascular adipose tissue (PVAT) (Figure 3 shows the inflammation observed in EAT and PVAT in four different patients). Aortic inflammation was associated with increased C-reactive protein (14.4 ± 32.8 mg/L, *n* = 23, vs. 2.86 ± 3.13 mg/L in patients without aortic inflammation, *n* = 8; *p* = 0.043), with higher plasma fibrinogen level (3.94 ± 1.01 mg/L, *n* = 26, vs. 3.4 ± 0.3 mg/L in patients without aortic inflammation, *n* = 7; *p* = 0.06), and with a higher BMI-corrected EATV (2.97 ± 1.62 cm^3^/(kg/m^2^) *n* = 11 vs. 1.03 ± 0.45 cm^3^/kg/m^2^, in patients without aortic inflammation, *n* = 5; *p* = 0.009), but not with inflammation in adipose tissues. Lastly, inflammation in the PVAT was correlated with inflammation in the epicardial adipose tissue (R = 0.67, *p* = 0.0001) but not with EATV (*p* = 0.15). In patients with CAD, CKD was not associated with a higher degree of inflammation in the aorta (85.7% in patients with CKD, *n* = 14, vs. 83.3% in patients without CKD, *n* = 18; *p* = ns) suggesting that the increase of mortality associated with CKD patients was not only related to inflammation in the artery.

**Figure 4:**
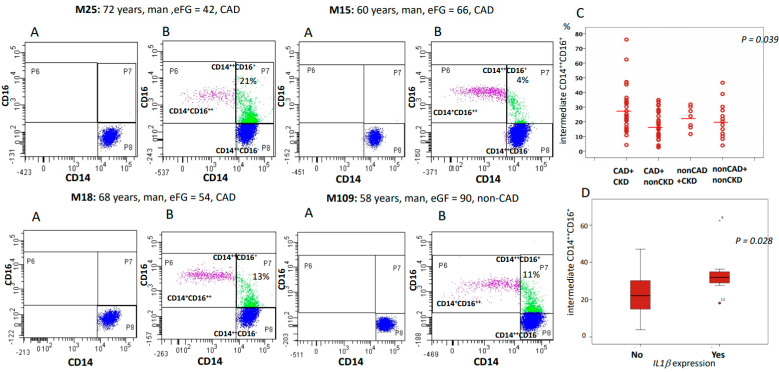
Flow cytometric analysis demonstrating human monocyte heterogeneity. Representative Flow cytometry plots for classical (CD14^++^CD16^−^), intermediate (CD14^++^CD16^+^) and nonclassical monocytes (CD14^+^CD16^++^) from four different patients. In all the cases, panels (**A**) represent the fluorescence minus one (FMO) negative control, while panels (**B**) represent monocyte subpopulations in the patient tested. Panel (**C**): dot-blot graphic showing the percentage of intermediate CD14^++^CD16^+^ monocytes from each study group. (**D**): Percentage of intermediate CD14^++^CD16^+^ monocytes according to the expression of *IL1β*. Abbreviations: CAD = coronary artery disease, NonCAD = patients without CAD, CKD = chronic kidney disease, NonCKD = patients with eFG > 60 mL/min. *p* value was calculated using the ANOVA test or U-Mann–Whitney test.

We were able to obtain optimal samples for cytometry from 77 patients. CAD patients with impaired renal function showed an increased proportion of intermediate CD14^++^CD16^+^ monocytes than patients with CAD and eGFR > 60 mL/min (*p* = 0.014) suggesting a role for renal function in the generation of inflammatory environments associated with ATH progression (Table 2 shows the distribution of monocytic subpopulations in patients with CAD and CKD). The analysis also revealed considerable interindividual variations (Figure 4C), but the underlying mechanisms for such variation are unknown. Furthermore, cell counts of intermediate CD14^++^CD16^+^ monocytes were correlated with levels of serum creatinine (*p* = 0.011, R = 0.29), and eGFR (*p* = 0.022, R = −0.27), and were higher in patients with aortic inflammation (28.4 ± 16.9%; *n* = 13) than in patients without aortic inflammation (15.3 ± 8.0%; *n* = 7; *p* = 0.043).

*IL1β* is the major circulating form of *IL1* with a subsequent downstream effect on *IL6* and has been proposed as a main contributor to ATH progression as well as a potential target to improve cardiovascular outcomes [17]. We next analyzed the expression of *IL1β*, as well as of a number of inflammatory cytokines (*IL6, TNFα*), chemokine receptors (*CXC3CR1, CCR5*), and MAP kinases (*MAPK1, MAP2K1, MAPK9*) in resting PBMCs from a subgroup of 36 patients (27 patients with CAD and 9 controls). *IL1β* was upregulated in 33.3% of patients with CAD (9 out of 27) but only in 11.1% of patients without CAD (1 out 9 patients) (*p* = 0.19), and in 32% of patients with CKD (8 out 25) but only in 18.2% of patients with eCFR > 60 mL/min (2 out 11) (*p* = 0.39). Taking in consideration this proportion, we would need two groups of 52 patients to detect a significant difference among CAD and nonCAD. The expression of *IL1β* was correlated with the expression of *IL6* (R = 0.9, *p* = 0.001), *TNFα* (R = 0.6, *p* = 0.05), and with the number of intermediate CD14^++^CD16^+^ monocytes (Figure 4) suggesting that the *IL1β/IL6* signaling pathway may be activated in proinflammatory CD16^+^ monocytes.

**Figure 5:**
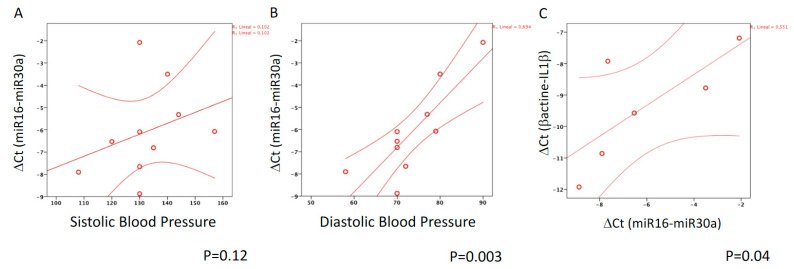
Expression of hsa-miR30a-5p was significantly correlated with diastolic blood pressure and with *IL1β* levels. Scatter plot showing the relationship between hsa-miR30a-5p and blood pressure (panels (**A**,**B**)) and *IL1β* expression (panel (**C**)). A straight line shows the linear trend and the correlation coefficient (R^2^). The Spearman’s rank order correlation was used.

Since high blood pressure has been correlated with inflammation, we also analyzed expression of hsa-miR-30a-5p, a known regulator of pro-inflammatory cytokine levels that has also been associated with a reduction of capillary network density and hypertension [18]. Hsa-miR-30a-5p was found to be expressed in PBMCs from eight out of 13 patients with CAD (61.5%) vs. only two out of six patients with nonCAD (33.3%; *p* = 0.2). Expression of hsa-miR-30a-5p was correlated with diastolic blood pressure (R = 0.832, *p* = 0.003) and with the expression of *IL1β* (R = 0.8, *p* = 0.042) (Figure 5), suggesting a link between microvascular network density and inflammation.

## 3. Discussion

In this study, we have observed that CKD patients with severe CAD disease showed a higher systolic blood pressure, a higher count of circulating proinflammatory CD14^++^CD16^+^ monocytes, and higher susceptibility to acute kidney injury (CSA-AKI) and death after CABG surgery than patients with eGFR > 60 mL/min, indicating that pathological pathways in atherogenesis progression might differ between patients with intact or impaired renal function. Furthermore, we also describe the association of aortic inflammation and serological markers of inflammation such as C-reactive protein and fibrinogen levels with the total volume of epicardial adipose tissue (EATV) and with increased counts of circulating, proinflammatory, intermediate CD14^++^CD16^+^ monocytes. Lastly, we observed that expression of hsa-miR-30a-5p, a negative regulator of microvascular network density and regulator of pro-inflammatory cytokine levels, was correlated with hypertension and with the expression of *IL1β*.

It is well known that CKD increases mortality and accelerates cardiovascular disease independently of the severity of the impairment of renal function [2], but the mechanisms involved remain largely undefined. Conversely, CKD is also an important risk factor for the development of AKI in patients with diabetes or hypertension [19]. Additionally, the reported prevalence of cardiac surgery–associated AKI is up to 20% and is associated with a high in-hospital mortality [20,21]. Thus, CKD and AKI are closely linked since AKI is associated with a higher risk of CKD as compared to the patients without AKI [22], and CKD is also an important risk for the development of AKI [23]. Pro-inflammatory cytokines’ upregulation or the decrease in their renal clearance in CKD may contribute to the accumulation of chemokines and inflammatory cells in the kidney, increasing the susceptibility to AKI [24,25]. In this study, we have confirmed that CKD is a risk factor for CSA-AKI since its incidence after CABG was of 26% in patients with CKD but only 5.2% in patients with eGFR > 60 mL/min. Similarly, in the control group without CAD, the incidence of AKI after valvular replacement surgery was 26.7% in patients with CKD but only 7.7% when eGFR was above 60 mL/min. CKD was also associated with a higher risk of death after CABG surgery (incidence of 15.7% in patients with CKD vs. 3.4% in patients with eGFR > 60 mL/min) or valvular replacement surgery (20% in CKD vs. 3.7% in patients with eGFR > 60 mL/min). These data support the impact of renal disease in the outcome of heart surgery patients. In our study, we observed that the inflammation in the aorta was associated with increased levels of inflammation markers (C-reactive protein and plasma fibrinogen level) but not with CKD. This result is not surprising since aortic samples were all obtained from patients during the CABG and ATH is characterized by a chronic, low-grade inflammatory response that attracts cells of the innate and adaptive immune systems into the plaque. These data suggest that the increase of mortality associated with CKD patients was probably not related to inflammation in the artery but associated with CSA-AKI.

Chronic inflammation is also a common feature in CKD patients due to multiple factors such as the induction of pro-inflammatory cytokines, oxidative stress, and uremia, a high risk of infection [26]. The upregulation of two inflammatory biomarkers (C-reactive protein and soluble tumor necrosis factor receptor II) and its association with the progression of CKD have been observed in a large cohort of patients with myocardial infarction–induced CKD [24]. In our study, patients with CKD displayed a higher systolic blood pressure, a higher proportion of intermediate CD14^++^CD16^+^ monocytes, and increased C-reactive protein levels than patients with eGFR > 60 mL/min. While several mechanisms contribute to the development of hypertension in CKD patients [27], a role of the immune system has also been proposed [28]. This chronic low-grade inflammation has been associated with ATH and plays an important role in the development of cardiovascular diseases [29]. Therefore, recruitment of monocytes into the vessel wall would contribute to the onset and progression of atherosclerosis. Alternatively, it has been also proposed that another potential source of monocyte activation is the interaction with the activated vascular endothelium [30]. Thus, high numbers of proinflammatory CD14^++^CD16^+^ monocytes were associated with the risk of cardiovascular disease and death in a cohort of 94 dialysis patients [7]. In our cohort of CKD patients, we observed that circulating proinflammatory CD14^++^CD16^+^ monocytes showed upregulated expression of *IL1β* in 22% of patients with CAD, which might also contribute to the endothelial damage and promote accelerated ATH and increased CVD risk. These data are interesting since several data implicate *IL1β* in the pathogenesis of ATH, and it has been used as a therapeutic target in the Canakinumab Anti-inflammatory Thrombosis Outcome Study (CANTOS) in about 10,000 patients who have sustained a prior myocardial infarction [31]. The study showed a significant 17% reduction of non-fatal MI, non-fatal stroke, or cardiovascular death. Our data suggest that only patients with upregulation of *IL1β* would get benefit from anti-*IL1β* therapy.

CKD induces alterations in the tissue microenvironment that may directly induce vascular pathology. The endothelial dysfunction in CKD leads to changes in microcirculation, adhesion of inflammatory cells, vascular permeability, and abnormal glomerular leakage [26]. The loss of the peritubular capillary contributes to the development of renal fibrosis and is associated with hypertension and proteinuria [32]. In addition, in a model of renal mass reduction, a strong association of tubulointerstitial fibrosis and glomerulosclerosis with modest blood pressure has been described [33]. Thus, even small increases of arterial pressure are transmitted directly to the glomeruli because of impaired glomerular autoregulation with progression of renal diseases. We have previously reported a decrease in renal microcirculation associated with ATH in a mice model [34]. Thus, we analyzed the expression of hsa-miR-30a-5p, a known negative regulator of microvascular network density and hypertension [18]. In our cohort of patients, hsa-miR-30a-5p expression was positively correlated with hypertension and with the expression of *IL1β*. These data suggest a link between microvascular network density and inflammation. Taking all data together, it is suggested that CKD associated with a reduction in renal microcirculation is also associated with higher inflammation that contributes to a higher risk of AKI and mortality after heart surgery.

This work has a number of limitations that could potentially hinder the significance of the results obtained. Firstly, this study involved a limited number of patients from a single center. Thus, some potential clinical biomarkers for CAD such as EATV may be not observed since a sample size of 165 patients has been estimated to be the minimum required to detect a correlation with CAD [35]. In our study, we analyzed 48 patients who had a CT scan performed as part of their pre-operative evaluation and observed a correlation with age and gender suggesting the presence of potential confounding factors. Secondly, the immune functions associated with monocytic subpopulations are puzzling, since functional redundancies between them have been observed with conflicting results. Lastly, interindividual variations of gene expression (i.e., in adhesion molecules) have been also identified that could impact on the pathological roles of the different subpopulations of monocytes [36].

Lastly, a number of future research directions with potential impact on practice can be drawn from this study:

1. CKD patients with severe CAD disease have higher susceptibility to acute kidney injury (CSA-AKI) and death after CABG surgery and require a deep evaluation prior to heart surgery.
2. The inflammation associated with CKD maybe one of the mechanisms responsible for the increase in the mortality of these patients.

## 4. Materials and Methods

### 4.1. Subjects

A prospective case–control study was conducted in adults with CAD and CKD undergoing elective coronary artery bypass graft (CABG). Controls included the following: (1) patients with CAD and eGFR > 60 mL/min (CAD + nonCKD) who underwent CABG, (2) patients with CKD without CAD who underwent heart valve surgery (nonCAD + CKD), and (3) patients without CAD or CKD who underwent heart valve surgery (nonCAD + nonCKD). The study was performed between June 2016 and July 2017 at the Bellvitge University Hospital and was approved by the ethics committee (PR149/14, approved on 19 May 2016). Full informed consent was obtained from all patients. Samples and data from patients included in this study were provided by the Biobank HUB-ICO-IDIBELL (PT17/0015/0024), integrated in the Spanish National Biobanks Network.

### 4.2. Definition of Variables

We considered all causes of operative mortality (death during the hospitalization or after discharge, but within 30 days of surgery), acute kidney injury, or prolonged postoperative stay. The eGFR (estimated glomerular filtration rate) was calculated from serum creatinine using the Chronic Kidney Disease Epidemiology Collaboration (CKD-EPI) equation [37]. CKD was defined as eGFR < 60 mL/min/1.73 m^2^ for more than 3 months irrespective of cause [38]. Severity of CKD was based on the eGFR number (stage 3: eGFR between 30 and 59 mL/min/1.73 m^2^, stage 4: eGFR = 15–29, stage 5: eGFR less than 15, and stage 5D for dialysis). Fasting venous blood glucose, serum cholesterol, C-reactive protein, fibrinogen, triglycerides, and hemogram were analyzed with standard methods. Diagnostics of cardiac surgery–associated AKI (CSA-AKI), defined as an elevation in serum creatinine to at least 1.5-fold from baseline (mean serum creatinine value during 6 months before hospitalization) within seven days after cardiac surgery, was obtained from the Electronic Health Record System of the Bellvitge Hospital. Diabetes mellitus was considered if a specific treatment was used. Body mass index (kg/m^2^), a measure of body fat, was calculated using the patient’s height and weight.

### 4.3. Reagents

In this work, we used the following reagents. Ficoll (SepMATE^TM^ PBMC Isolation Tubes, StemCell technologies, Vancouver, BC, Canada), Maxwell RSC miRNA Tissue Kit (Cat. # AS1460, Promega, Madison, WI, USA), Maxwell RSC miRNA Plasma and Serum Kit (Cat. # AS1680, Promega, Madison, WI, USA), TaqMan^®^ Advanced miRNA Assays–single tube assays (Cat. #A25576, ThermoFisher, Waltham, MA, USA), TaqMan^®^ Advanced miRNA cDNA Synthesis Kit (Cat. # A28007, ThermoFisher, Waltham, MA, USA). Furthermore, the following TaqMan Gene Expression Assays (ThermoFisher, Waltman, MA, USA) were used to measure the expression of different genes:

*IL1α* (Hs_00174092_m1),
*IL6* (Hs_00174131_m1),
*TNFα*(Hs_00174129_m1),
*CCR5* (Hs_00152917_m1),
*Cx3CR1* (Hs_01922583_m1),
*MAPK1* (Hs_01046830_m1),
*MAP2K1* (Hs_05512159_s1),
*MAPK9* (Hs_01558224_m1)
*ACTIN* (Hs_01060665-g1)
hsa_miR30a-5p (Assay ID:000417)
hsa-miR16-3p (Assay ID:002171), and
cel-miR-54-3p (Assay ID:001361).

### 4.4. Measurement of the Epicardial Adipose Tissue Volume (EATV)

In all, 24 patients underwent a non-contrast cardiac computed tomography to assess the extent of coronary calcification by the Agatston coronary calcium score (CCS) in accordance with local clinical practices. We measured the total epicardial adipose tissue volume in a semi-quantitative manner by tracking the contour of the pericardium from the level of the pulmonary artery bifurcation to the apex of the heart at the most caudal end (Figure 1) and measuring with the open source DICOM (Digital Imaging and Communications in Medicine) medical image viewer Horos^TM^ (Horosproject.org). A minimum of 40 images was used, and technical parameters were adjusted based on the attenuation histogram of perivascular fat within the threshold range of −30 to −250 Hounsfield units. Fat volume was measured in cm^3^ (EATV) and was also indexed to BMI (EATV/BMI). EATV was measured blindly by two observers.

### 4.5. Specimen Details

An aorta sample and two additional samples from local PVAT were removed from patients during the surgery and collected in standard physiologic serum. PVAT samples corresponded to the surrounds of a damaged coronary artery and to another epicardial adipose tissue. Samples were sliced in two parts, and one was snap frozen while and the other one was fixed with formalin, embedded in paraffin, sectioned at 5 µm, and stained with hematoxylin/eosin. Aorta samples were considered optimal for analysis of inflammation if the intima of the vessel could be identified. Tissue samples were scanned at 20× magnification to generate a digital image of the section. Severity of vascular inflammatory lesions were evaluated using a semi-quantitative scoring system based in the mononuclear infiltration of the intima. The degree of inflammation was graded as “No arteritis” when <5 cells/field were observed, “mild arteritis” when there were 5−10 cells/field, “moderate arteritis” when there were 10−20 cells/field, and “severe intimal arteritis” when there were >20 cells/field (Figure 2).

### 4.6. Measurement of Adipocyte Size and Inflammation

The border of 10 intact adipocytes contained within the field of view in 20× magnification sections of PVAT samples was traced and measured using the Fiji image processing package v 2.1.0/1.53c [39]. Five sections were imaged from each patient. The extent of adipose tissue inflammation was evaluated taking in consideration the number of mononuclear cells. The degree of inflammation (Figure 3) was scored as absent (score 0) when less than two inflammatory cells/adipocytes were detected, 1 when greater than minimal, or 2 when severe capillary hyperemia was observed.

### 4.7. Flow Cytometry Analysis

Peripheral blood was collected before surgery into K_3_EDTA BD Vacutainer tubes (BD Biosciences, San Jose, CA, USA) and kept at room temperature until preparation for flow cytometry within 4 h. In total, 50 µL of whole blood was added to each flow-cytometry tube with appropriate amounts of fluorochrome-labeled mAbs (as determined after titration of the dose recommended by the manufacturers). The following antibodies were used in this study: anti-CD14^APC^ (Miltenyi Biotec, Bergisch Gladbach, Germany), anti-CD16^FITC^ (ThermoFisher, Waltham, MA, USA). Data were collected on a BD FACSCANTO II flow cytometer (BD Biosciences, San Jose, CA, USA) and analyzed with the BD FACSDiva Software v6.1.3 (BD Biosciences, San Jose, CA, USA).

### 4.8. Identification Strategy of Monocyte Subsets

Monocyte subpopulations were identify using an inclusion gating strategy [40]. Briefly, cells were first visualized on FSC vs. SSC, and an ample gate was drawn around the monocyte population excluding the majority of lymphocytes, neutrophils, and debris. Stepwise gating of daughter populations was performed to exclude the remaining neutrophils, NK/NKT-cells, T-cells, and B-cells. The resultant population was visualized on a plot comparing CD14 and CD16, and subsequently the subsets of CD14^++^CD16^−^, CD14^++^CD16^+^, and CD14^+^CD16^+^ were defined. A CD16 fluorescence-minus-one (FMO) control was used to determine the border between classical [CD14^++^/CD16^−^] and intermediate [CD14^++^/CD16^+^] monocytes. Finally, we subdivided CD16^+^ monocytes into functionally distinct intermediates CD14^++^CD16^+^ and non-classical CD14^+^CD16^+^ cells using a rectangular gate and aligning the base of the intermediates with the bottom of the concentric circles within the classical population [41]. Gates were adjusted for each sample, and results showed the proportion of each monocyte subset (Figure 4).

### 4.9. Total RNA Isolation and Quantitative Real-Time PCR

PBMCs were isolated less than 2 h after blood collection by Ficoll fractioning (SepMATE^TM^ PBMC Isolation Tubes, StemCell technologies, Vancouver, BC, Canada). Samples from aorta and perivascular adipose tissue were extracted during the intervention when it was feasible. Total RNA was extracted using the Maxwell RSC miRNA Tissue KIT (Promega, Madison, WI, USA) and cDNA templates synthesized with the TaqMan^®^ Advanced miRNA cDNA Synthesis Kit (Cat# A28007, Thermofisher, Waltham, MA, USA). All real-time quantitative PCRs were performed by triplicate on a 7900HT Fast Real-Time PCR Instrument (ThermoFisher, Waltham, MA, USA) and using TaqMan^®^ Fast Advanced Master Mix. Expression analysis was performed using the comparative CT (ΔΔCT) method using β-actin as an endogenous control for normalization. Hsa-miR-30a-5p expression was normalized using hsa-miR-16-3p as an endogenous reference gene and 100 pmol/mL of exogenous cel-miR-54.

### 4.10. Statistics

Continuous variables are presented as means (SD). The proportion of deaths in surgical patients with CKD was analyzed with a chi-square test. To check the association of intermediate CD14^++^CD16^+^ with serum creatinine or eGFR, patients on dialysis therapy were excluded and a correlation of Spearman was performed. The correlation of has-miR30a with blood pressure, inflammatory cytokines, chemokines, and MAPKs was performed with Spearman’s rank order correlation. The U-shaped relationship of age and epicardial fat volume was analyzed using a quadratic regression model. Normality of variables was tested with the Kolmogorov test. ANOVA test with test of Levene for equal variances was used to compare normal variables, and nonparametric Kruskal–Wallis test when variables were not normally distributed. Differences were considered significant at *p* < 0.05. The different analysis was performed using SPSS v20 (IBM SPSS Statistics, www.ibm.com).

## 5. Conclusions

We have identified biomarkers linking CKD and CAD that might improve the current understanding of how an impaired kidney function leads to higher cardiovascular risks, as well as help to identify novel therapeutic targets. Our data suggest that only patients with upregulation of IL1β would get benefit from anti-IL1β therapy.

Key points of our study:

1. CKD is a risk factor for CSA-AKI and death after cardiac surgery.
2. CKD patients with CAD present a proinflammatory environment that can be detected in the blood (increased C-reactive protein, fibrinogen levels, intermediate CD14^++^CD16^+^ monocytes, and IL1β) and could be useful to stratify the cardiovascular risk of patients with CKD.
3. We suggest a link between microvascular network density and inflammation since we observed a link between has-miR30a-5p and IL1β expression.

## Figures and Tables

**Table 1:** Baseline clinical characteristics of patients.

	CAD + CKD	CAD + NonCKD	NonCAD + CKD	NonCAD + NonCKD	*p*
*n*	51	59	15	26	
Gender (F/M)	12/39	13/46	3/12	9/17	0.6
Age (years)	70 (10)	66(9)	71 (9)	63 (10)	0.003
BMI (kg/m^2^)	27.8 (4)	28.3 (3)	29.1 (4)	29 (5)	0.6
Cr (µmol/L)	239 (212)	81 (14)	192 (171)	73 (15)	0.0001
Diabetes (yes/no)	32/19	32/27	5/10	9/17	0.05
Cholesterol (mmol/L)	4.1 (1.5)	4.2 (1.4)	4.5 (1.4)	4.8 (1.2)	0.14
Triglycerides	1.9 (1.1)	2.03 (0.9)	2.03 (0.9)	2.9 (2.05)	0.25
SBP (mmHg)	129 (21)	123 (13)	121 (20)	119 (11)	0.02
DBP (mmHg)	70 (9)	69 (10)	69 (11)	68 (9)	0.6

Abbreviations: CAD = coronary artery disease, NonCAD = patients without CAD, CKD = chronic kidney disease, NonCKD = patients with eGFR > 60 mL/min, BMI = body mass index, Cr = creatinine, SBP = systolic blood pressure, DBP = diastolic blood pressure. *p* value was calculated using the Kruskal–Wallis nonparametric test and the chi-square test for frequencies. Standard deviations are shown in parentheses.

**Table 2:** Distribution of monocyte subpopulations in the peripheral blood of the patients included in this study.

	CAD + CKD	CAD + NonCKD	NonCAD + CKD	NonCAD + NonCKD	*p*
*n*	27	30	7	13	
CD14^++^CD16^−^	65.0 (17.8)	71.2 (12.5)	71.0 (7.7)	72.1 (13.2)	0.25
CD14^++^CD16^+^	28.2 (16.1)	18.5 (9.8)	22.6 (7.6)	21.2 (12.2)	0.039
CD14^+^CD16^++^	6.8 (3.9)	10.3 (9.1)	6.4 (6.2)	6.7 (6.6)	0.14
CD16^+^	35 (15.6)	28.8 (12.7)	29 (7.8)	28 (15.2)	0.45

Cell isolation and the gating strategy to detect the different monocyte subpopulations have been described in the methods section. Abbreviations: CAD = coronary artery disease, NonCAD = patients without CAD, CKD = chronic kidney disease, NonCKD = patients with eGFR > 60 mL/min. *p* value was calculated using the ANOVA test. Standard deviations are shown in parentheses.

## Abbreviations

ATH	Atherosclerosis
CABG	Coronary Artery Bypass Graft
CAD	Coronary Arterial Disease
CKD	Chronic Kidney Disease
CSA-AKI	Cardiac Surgery–Associated Acute Kidney Injury
EATV	Epicardial Adipose Tissue Volume
eGFR	estimated Glomerular Filtration Rate
FSC	Forward Scatter
ITD	Intermediate CD14^++^CD16^+^ monocytes
LPS	Lipopolysaccharide
MAP	Mitogen-Activated Protein Kinase
NK	Natural Killer cells
NKT	Natural Killer T cells
PBMCs	Peripheral Blood Mononuclear Cells
PVAT	Perivascular Adipose Tissue
SSC	Side Scatter
